# Concurrent Circulation of Viral Agents in Pediatric Patients Presenting with Respiratory Illness and Diarrheal Symptoms in Metropolitan Region of São Paulo, Brazil, 2021

**DOI:** 10.3390/v17040497

**Published:** 2025-03-29

**Authors:** Adriana Luchs, Natanael Sutikno Adiwardana, Leonardo Cecilio da Rocha, Ellen Viana, Simone Guadagnucci, Adriana Parise, Vanessa Cristina Martins Silva, Lais Sampaio de Azevedo, Raquel Guiducci, Yasmin França, Natacha Luana Pezzuol Frank, Ana Lucia Nascimento da Silva, Andre Luiz Vianna de Oliveira, André Henrique Souza Azevedo, Bárbara Segatelli Carreteiro, Maurício Lacerda Nogueira

**Affiliations:** 1 Virology Center, Adolfo Lutz Institute, Sao Paulo 01246-902, Brazil; 2 São José do Rio Preto School of Medicine (FAMERP), São José do Rio Preto 15090-000, Brazilmauricio.nogueira@edu.famerp.br (M.L.N.); 3 Infection Prevention and Control Service, Barueri Central Emergency Center, Barueri 06401-000, Brazil; nateldiw@yahoo.com.br (N.S.A.);; 4 Pediatric Emergency Care, Barueri Central Emergency Center (SAMEB), Barueri 06401-000, Brazil; 5 Department of Pathology, University of Texas Medical Branch, Galveston, TX 7555-0609, USA

**Keywords:** viral infections, gastro-respiratory illness, co-infection, syndromic surveillance, viral diversity

## Abstract

Pneumonia and diarrhea are the leading causes of death in children under 5 globally, worsened by viral infections. This study investigates viral agents in children ≤ 3 years with respiratory illness and diarrhea in Metropolitan Region of São Paulo, Brazil, during spring 2021. Twenty paired samples (oropharyngeal swab and feces) were tested using in-house qPCR for HBoV and HAdV, RT-qPCR for RVA, EV, PeV-A, and NoV, and a commercial RT-qPCR kit for SARS-CoV-2, Flu A/B, and RSV. HAstV was detected with conventional nested (RT)-PCR. Positive samples were sequenced for molecular characterization and phylogenetic analysis. Seven viruses were identified: HBoV, NoV, HAdV, PeV-A, EV, RSV, and Flu A. HBoV and NoV were detected in 75% of cases, with co-infection in 65% of patients, indicating their involvement in the gastro-respiratory illness. Genotyping of HBoV (HBoV-1), NoV (GII.4_Sydney[P16], GII.2[P16], and GII.4_Sydney[P31]), EV (Coxsackievirus A6), HAdV (species C, type 6), and PeV-A (genotype 1) showed local virus diversity. Phylogenetic analysis indicated no ongoing community outbreak, with distinct clusters observed. The findings highlight the overlap of respiratory and enteric diseases, revealing local viral diversity and high exposure to enteric viruses. This underscores the challenges in differential diagnosis and the need for syndromic surveillance.

## 1. Introduction

Pneumonia and diarrhea are leading causes of death in children under age 5 worldwide, resulting in 1.23 million annual fatalities (https://www.jhsph.edu/ivac/wp-content/uploads/2020/11/IVAC_PDPR_2020.pdf accessed on 28 February 2025). Viral infections causing diarrhea have posed a significant public health challenge over decades, yet clinical presentations often lack clear indications of specific viral agents [1]. Group A rotaviruses (RVA), noroviruses (NoV), enteric adenoviruses (HAdV), and astroviruses (HAstV) are commonly identified as enteric viral pathogens in cases of acute gastroenteritis [1].

Gastrointestinal symptoms are also observed in various respiratory virus infections, such as influenza and severe acute respiratory syndrome coronavirus 2 (SARS-CoV-2) [2]. These symptoms may arise due to co-infections involving respiratory and enteric viruses during concurrent outbreaks of respiratory infections and gastroenteritis, potentially linking symptoms that could mislead clinical assessments [3]. Enteroviruses (EVs) are widely associated with respiratory diseases, acute flaccid paralysis, aseptic meningitis, encephalitis, hand-foot-and-mouth disease, febrile illnesses, neonatal sepsis, and cardiopathies [4]. While EVs have been sporadically linked to diarrhea in some studies, their definitive role in causing human gastroenteritis remains uncertain [5]. Emerging viruses like human bocaviruses (HBoV) and Parechoviruses A (PeV-A) have also been reported to be associated with both acute gastroenteritis and respiratory diseases [6,7].

Surveillance is crucial for public health, providing essential data for guiding interventions. However, there remains a lack of comprehensive data on the syndromic landscape of viral agents associated with respiratory and diarrheal illnesses in Brazil. Most studies have focused primarily on diagnosing single viral infections (e.g., RVA or NoV) in cases of acute diarrhea or specific viruses such as Influenza A/B (Flu A/B), SARS-CoV-2, and Respiratory Syncytial Virus (RSV) in investigations of Severe Acute Respiratory Illness [8,9]. Implementing syndromic surveillance that includes testing for newly emerging viruses could enhance understanding of the spectrum of viral agents linked to acute gastroenteritis and respiratory diseases in Brazil. This approach could particularly advance differential diagnosis in cases presenting with combined gastrointestinal and respiratory symptoms.

In this study, we identified and molecularly characterized viral agents causing acute respiratory disease in children ≤ 3 years old presenting with diarrheic symptoms in the northwestern region of the Metropolitan Region of São Paulo, Brazil, during spring 2021. Phylogenetic analysis was conducted to explore genetic relationships with previously reported viruses.

## 2. Material and Methods

### 2.1. Study Population

A significant increase in children under 3 years of age presenting with both respiratory and gastrointestinal symptoms was noted at the Pediatric Emergency Care unit of Barueri Medical Assistance Service (SAMEB) in mid-August 2021. With negative COVID-19 tests, the possibility of an emerging virus (e.g., HBoV or PeV-A) was considered. Therefore, the present study included children with enteric and respiratory symptoms whose parents provided the Informed Consent Form. After three months, recruitment was halted as the number of cases decreased. It is important to highlight that limited staff availability in the overwhelmed COVID-19 emergency room hindered further recruitment.

This case report was conducted from August 30th to December 6th, 2021, involving 20 children aged ≤ 3 years who presented with respiratory and diarrheic symptoms at SAMEB. The study utilized secondary data from medical records collected during routine care, ensuring patient confidentiality through the anonymization of all information. Basic demographic details (age, gender) and clinical information (symptom onset and collection dates, symptoms like cough, diarrhea, vomiting, fever, and runny nose) were extracted from clinical records (Table 1). 

Oropharyngeal swabs and fecal samples collected from each patient were stored at −20 °C and later screened for viruses at the Research Laboratory of Virology, Faculty of Medicine of São José do Rio Preto (FAMERP). SAMEB, affiliated with the Municipality of Barueri’s Health Department, operates under Brazil’s Unified Health System (Sistema Único de Saúde—SUS) and serves as a key healthcare facility in the northwest region of São Paulo municipality, Brazil. Barueri is ranked as the 14th wealthiest city in Brazil, with a population of 316,473 residents spread over 65.70 km^2^ (https://www.ibge.gov.br/cidades-e-estados/sp/barueri.html accessed on 28 February 2025).

### 2.2. Viral Screening

Viral DNA/RNA from oropharyngeal and 10% fecal samples was extracted using the Zymo Quick-DNA/RNATM Viral MagBead kit (Zymo Research^®^, Orange, CA, USA) following the manufacturer’s instructions. Screening for RVA, EV, NoV, and PeV-A utilized reverse transcription quantitative real-time PCR (RT-qPCR) with established primers and protocols [10,11,12,13]. HAdV and HBoV detection employed quantitative real-time PCR (qPCR) as described by Heim et al. [14] and Lu et al. [15], respectively. SARS-CoV-2, Flu A, Flu B, and RSV were detected using the Allplex^TM^ SARS-CoV-2/FluA/FluB/RSV RT-qPCR kit (Seegene Inc., Seoul, Republic of Korea) following manufacturer guidelines. Samples were considered positive at Ct values ≤ 39. Reverse transcription used random primers, and HAstV detection in cDNA employed heminested PCR targeting the RdRp gene (422 bp) with protocols by Chu et al. [16].

### 2.3. HAdV, HBoV, NoV, EV, and PeV-A Typing

Positive HAdV, HBoV, EV, NoV, and PeV-A samples underwent molecular characterization. Viral DNA/RNA from oropharyngeal swabs and/or feces was directly amplified and sequenced using Sanger sequencing for phylogenetic analysis.

HAdV strains were genotyped using hexon gene-specific primers (Ad1 and Ad2) targeting a 482-bp section, following Xu et al.’s protocol [17]. HBoV samples were amplified using VP1/VP2 region-specific primers in a conventional PCR method by Durigon et al. [18], yielding a 657-bp amplicon.

NoV samples were amplified using MON 431 and G2SKR primers targeting a 570-bp fragment covering regions B and C (3′-end of ORF1 and 5′-end of ORF2) [19] with the SuperScript III One-Step RT-PCR System (Invitrogen, Life Technologies, Carlsbad, CA, USA).

EV and PeV-A samples underwent conventional RT-PCR with random primers. EV VP1 region-specific primers were used in a semi-nested PCR following Iturriza-Gómara et al.’s method [20], while PeV-A samples were amplified using nested PCR targeting the VP3/VP1 junction region according to Ghanem-Zoubi et al.’s procedure [21].

PCR products were analyzed on 1.5% agarose gel with GelRed™ and a 100-bp molecular size ladder. Sequencing was performed using the BigDye™ Kit v3.1 (Applied Biosystems, Foster City, CA, USA) and ABI 3130 sequencer (Applied Biosystems, Foster City, CA, USA) at Premium Network of Multi-User Equipment/IMT/FMUSP. Chromatograms were analyzed with Sequencher^TM^ 4.7 software (Gene Codes Corporation, Ann Arbor, MI, USA). Genotype assignment utilized Basic Local Alignment Search Tool BLAST (http://blast.ncbi.nlm.nih.gov, accessed on 24 March 2025), Genoma Detective (https://www.genomedetective.com/, accessed on 24 March 2025), Human Calicivirus Typing (HuCaT) (https://calicivirustypingtool.cdc.gov/bctyping.cgi, accessed on 24 March 2025), and Enterovirus Typing Tool Version 0.1 (https://www.rivm.nl/mpf/typingtool/enterovirus, accessed on 24 March 2025).

### 2.4. Phylogenetic Analysis

The sequences obtained were aligned with prototype sequences of HBoV, NoV, EV, PeV-A, and HAdV retrieved from GenBank using CLUSTAL W in BioEdit Sequence Alignment Editor Software version 7.0.5.2 (Ibis Therapeutics, Carlsbad, CA, USA). Maximum likelihood trees were constructed for each pathogen using MEGA X software [22] with substitution models selected based on the Akaike Information Criterion (AICc). The models used were Tamura-Nei (TN93)+I for HBoV-1, Kimura 2-parameter (K2)+G+I for NoV ORF1, K2+G for NoV ORF2, Hasegawa-Kishino-Yano (HKY)+I for HAdV-C6, K2+G+I for CVA6, and Tamura 3-parameter (T92)+G+I for PeV-A1. Statistical significance for each branching point was assessed with 1000 replicates.

## 3. Results

### 3.1. Clinical Findings

Table 1 summarizes clinical findings from 20 patients in the study. The mean and median ages were 1.8 years (range: 11 days to 3 years) and 1 year, respectively. Males accounted for 60% (12/20) of the samples, while females made up 40% (8/20). Diarrhea was the predominant symptom (85%, 17/20), followed by vomiting (60%, 12/20). Fever and coryza were observed in 45% (9/20) and 40% (8/20) of patients, respectively. Less frequently reported symptoms included cough, abdominal pain, productive cough, sore throat, and sneezing. No severe outcomes were reported.

### 3.2. Viral Diagnosis

Seven different viruses were identified: HBoV, NoV, HAdV, PeV-A, EV, RSV, and Flu A. HBoV and NoV were found in 75% (15/20) of cases, with co-infections observed in 13 patients (65%). These findings suggest their involvement in respiratory illness with gastrointestinal symptoms at SAMEB. Due to limited sample volume, oropharyngeal swabs from three cases (N05, N06 and N07) were excluded from extraction, leaving only fecal samples for analysis. HBoV was detected in both sample types from eight cases, while NoV was exclusively found in stool specimens (Table 1).

HAdV, EV, PeV-A, and RSV were detected in two cases each (10%, 2/20) and Flu A in one case (5%, 1/10). EV was detected in both paired samples (oropharyngeal swab and fecal sample) in positive cases. RSV was found in one case from paired samples and in another case from the swab alone. HAdV and PeV-A were identified exclusively in fecal samples, while Flu A was found only in oropharyngeal swabs. All samples tested negative for RVA, HAstV, and SARS-CoV-2 (Table 1).

Co-infections involving respiratory and enteric viruses were notably high (75%, 15/20). The most common co-infection was HBoV+NoV (40%, 8/20), followed by triple infections including HBoV+NoV+RSV (10%, 2/20), HBoV+NoV+HAdV (10%, 2/20), and others like NoV+PeV-A (5%, 1/20), HBoV+NoV+EV (5%, 1/20), and NoV+EV+PeV-A (5%, 1/20) (Table 1).

### 3.3. Genotypic Data

Out of 23 HBoV-positive specimens (11 oropharyngeal swabs and 12 fecal samples), 10 (43.5%) were successfully genotyped as HBoV-1. NoV-positive samples exhibited significant genetic diversity; all 15 were classified as genogroup II by RT-qPCR. Sanger sequencing of partial RNA polymerase and capsid regions was successful for 13 samples. The predominant genotype was NoV GII.4_Sydney[P16] (69.2%, 9/13), followed by GII.2[P16] (27.1%, 3/13) and one GII.4_Sydney[P31] strain (7.7%, 1/13). Four sequenced EV samples (two oropharyngeal swabs and two stool samples) were identified as Coxsackievirus A6 (CVA6). Two fecal HAdV-positive samples were identified as HAdV-C6. Of two PeV-A-positive fecal samples, one was genotyped as PeV-A1 (Table 1).

### 3.4. Phylogenetic Data

**Figure 1:**
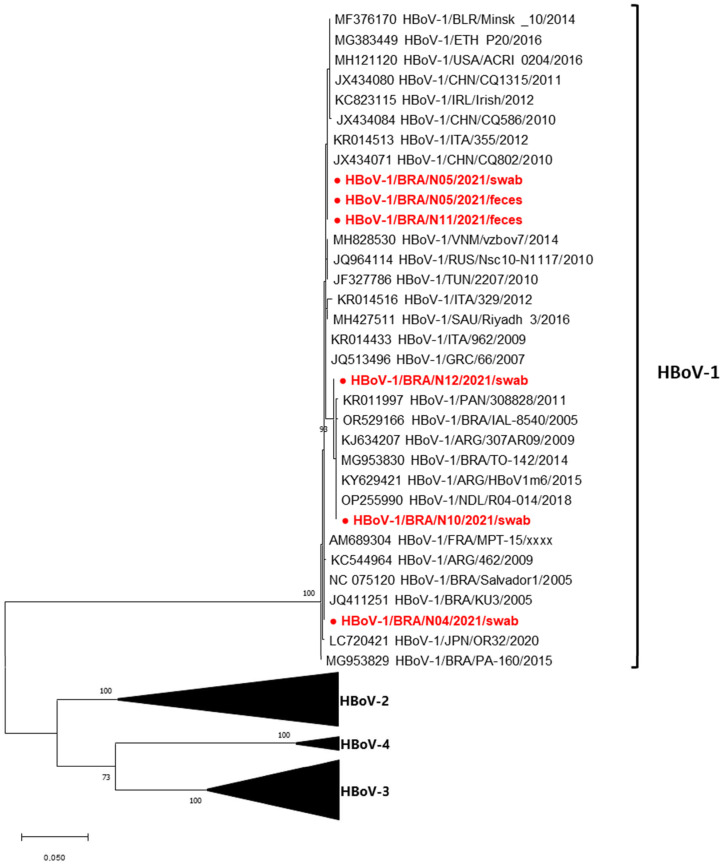
Maximum likelihood phylogenetic tree of the partial VP1/VP2 HBoV-1 protein nucleotide sequence (658-bp fragment) generated with MEGA X software [22] of the Brazilian HBoV-1 strains detected from children with acute respiratory disease and diarrheic symptoms, Barueri, São Paulo, Brazil, 2021 (highlighted in red). HBoV prototype strains were obtained from GenBank database. HBoV assigned genotypes are indicated on the right. Accession numbers of each strain are displayed. The scale indicates the number of divergent nucleotide residues. The percentage of bootstrap values is shown at the branch node.

High-quality sequences were selected as representative HBoV strains for phylogenetic analysis. The phylogenetic tree indicated distinct clustering of Brazilian HBoV-1 strains, implying that the HBoV-1 strains associated with respiratory illnesses and gastrointestinal symptoms in children treated at SAMEB do not originate from the same source. Brazilian HBoV-1 sequences showed 99–100% nt among each other. Strain N04/swab grouped with strains from Brazil, Argentina, France, and Japan (2005–2020, 100% nt). Strains N10/swab and N12/swab clustered with those from Brazil, Argentina, Panama, and the Netherlands (2005–2018, 100% nt). Strains N05/swab, N05/feces, and N11/feces clustered with strains from America, Europe, Africa, and Asia (2010–2016, 100% nt) (Figure 1).

**Figure 2:**
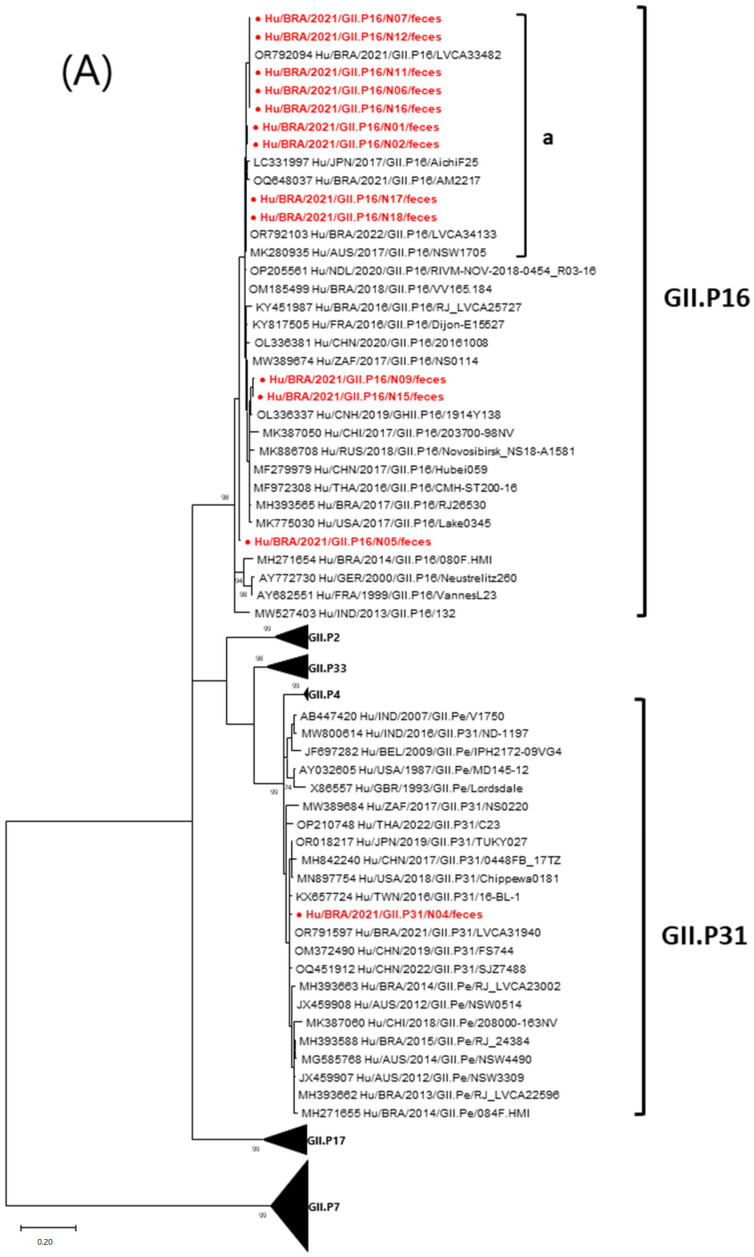

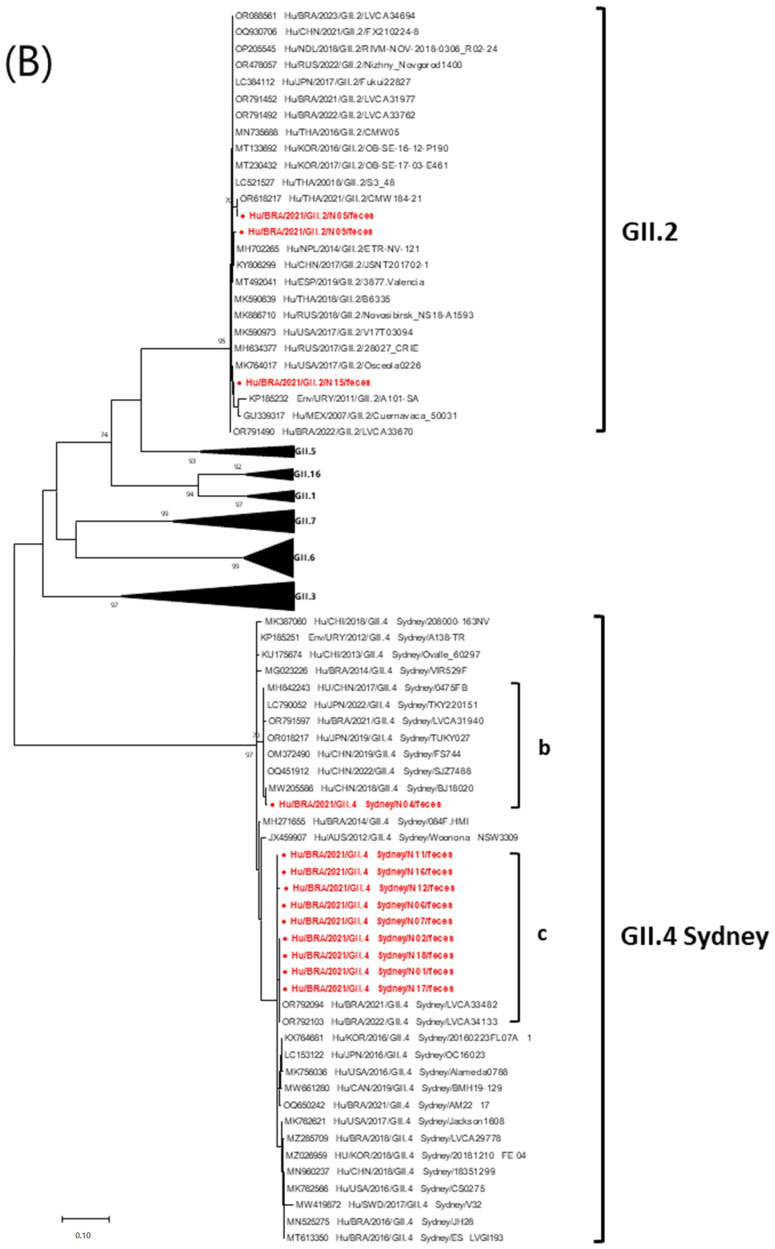
Phylogenetic analysis of Brazilian NoV strains detected from children with acute respiratory disease and diarrheic symptoms, Barueri, São Paulo, Brazil, 2021 (highlighted in red) alongside other selected NoV strains. Maximum-likelihood trees are presented for partial ORF 1 (**A**) and ORF 2 (**B**). Reference NoV sequences were retrieved from the GenBank database, with each strain’s accession number, isolate, country, and year indicated. Clusters identified in the present study are denoted by letters a, b, and c. The NoV G-types (ORF2) and P-types (ORF1) are specified. Statistical significance for each branch point was determined using 1000 pseudo-replicate datasets. The scale bar represents nucleotide substitutions per site, and bootstrap values are displayed as percentages at the branch nodes.

Phylogenetic trees were constructed using the partial RNA polymerase gene region (282 bp) and capsid region (270 bp) of NoV GII strains. The GII.4_Sydney[P16] ORF2 sequences exhibited 99.1–100% nt and grouped in cluster “c” with other Brazilian sequences from 2021–2022 (Figure 2B). The ORF1 region analysis showed that Brazilian [P16] sequences paired with GII.4 (99.1–100% nt) had a notable affinity with Brazilian strains from 2021–2022 and the Australian strain NSW1705 from 2017 (cluster “a”) (Figure 2A). The N04 GII.4_Sydney[P31] strain formed a separate group “b”, closely related (99.2–99.4% nt) to Asian and Brazilian sequences from 2017–2021 (Figure 2B). The N04 [P31] ORF1 sequences showed 100% nt with the Brazilian GII.4_Sydney[P31] LVCA31940 strain from 2021 (Figure 2A). Brazilian GII.2[P16] sequences demonstrated 98.4–99.5% nt in ORF1 and 98.5–100% nt in ORF2 with global strains from 2016–2023, and 97.8–100% nt among themselves across both ORFs (Figure 2A,B).

**Figure 3:**
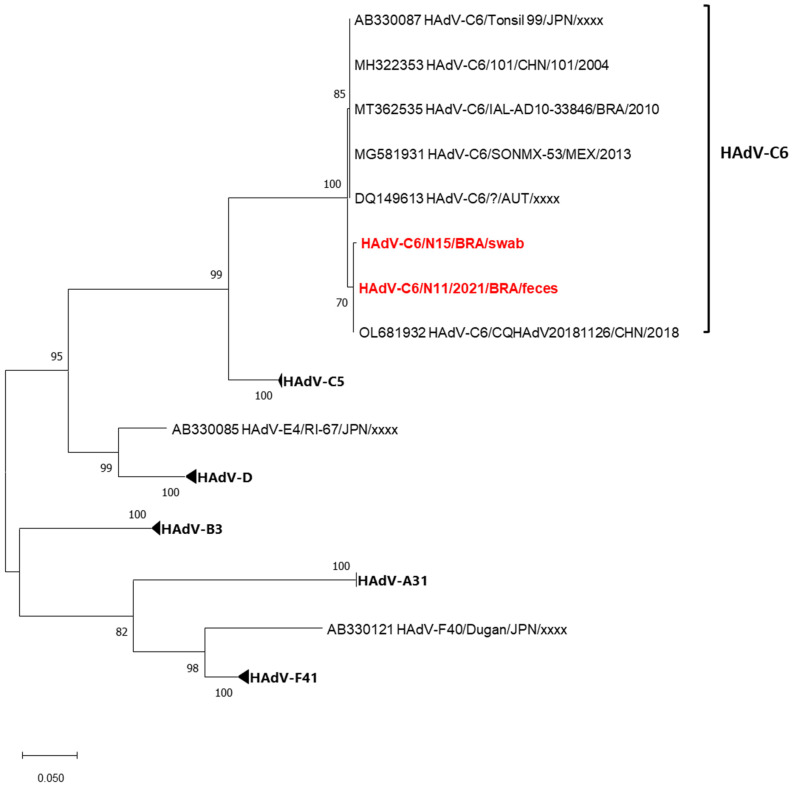
Maximum likelihood phylogenetic tree of the partial Hexon nucleotide (449-bp fragment) generated with MEGA X software [22] of the Brazilian HAdV-C6 strains detected from children with acute respiratory disease and diarrheic symptoms, Barueri, São Paulo, Brazil, 2021 (highlighted in red). HAdV prototype strains were obtained from GenBank database. HAdV assigned genotypes are indicated on the right. Accession numbers of each strain are displayed. The scale indicates the number of divergent nucleotide residues. The percentage of bootstrap values is shown at the branch node.

The Brazilian HAdV-C6 sequences from N15/feces and N11/swab were closely related, showing 99.8% nt, indicating a common origin. These strains exhibited the highest homology (99.8–100% nt) with the CQHAdV20181126 strain from China, identified in 2018 (Figure 3).

**Figure 4:**
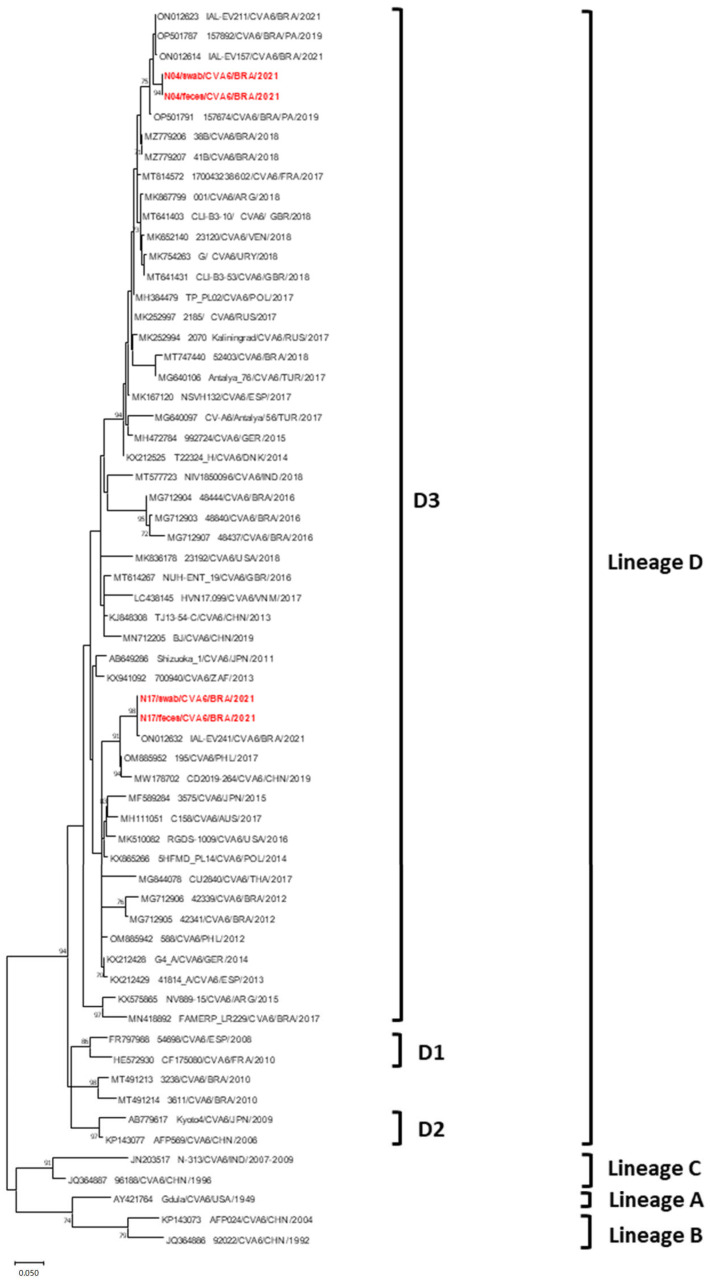
Maximum likelihood phylogenetic tree of the partial VP1 protein nucleotide (334-bp fragment) generated with MEGA X software [22] of the Brazilian CVA6 strains detected from children with acute respiratory disease and diarrheic symptoms, Barueri, São Paulo, Brazil, 2021 (highlighted in red). CVA6 prototype strains were obtained from GenBank database. CVA6 assigned lineages are indicated on the right. Accession numbers of each strain are displayed. The scale indicates the number of divergent nucleotide residues. The percentage of bootstrap values is shown at the branch node.

The Brazilian CVA6 N04 (feces and swab) and N17 (feces and swab) samples were assigned to clade D, sub-lineage D3. The CVA6 lineages (A–D) were classified according to previous proposals [23]. Strains N04/feces and N04/swab (identical sequences) clustered with Brazilian strains from 2019–2021 (98.4–99% nt). Strains N17/feces and N17/swab (identical sequences) grouped with Brazilian strains from 2021 (99.7% nt) and Asian strains from 2017–2019 (95.7–98.1% nt). N04 and N17 strains are remotely related (89.6–89.7% nt), suggesting different origins (Figure 4).

**Figure 5:**
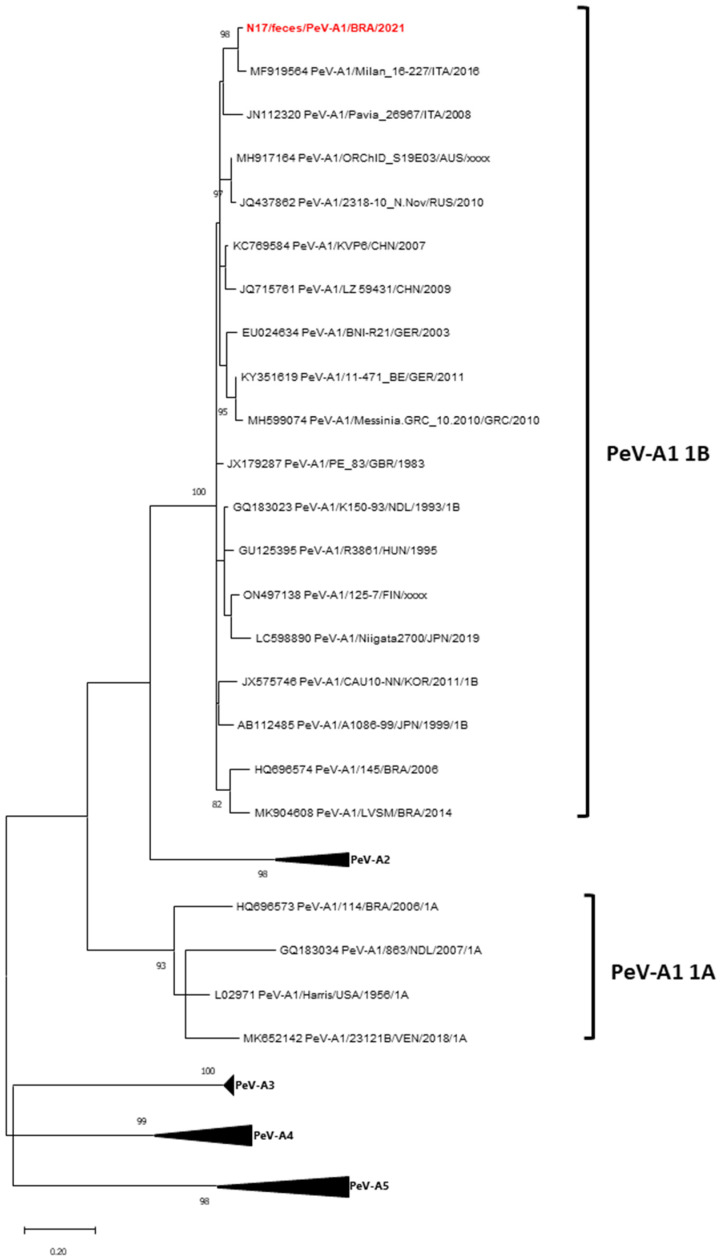
Maximum likelihood phylogenetic tree of the partial VP3/VP1 junction region (280-bp fragment) generated with MEGA X software [22] of the Brazilian PeV-A1 strain detected from children with acute respiratory disease and diarrheic symptoms, Barueri, São Paulo, Brazil, 2021 (highlighted in red). PeV-A prototype strains were obtained from GenBank database. PeV-A1 assigned subtypes are indicated on the right. Accession numbers of each strain are displayed. The scale indicates the number of divergent nucleotide residues. The percentage of bootstrap values is shown at the branch node.

Phylogenetic analysis of the partial VP1/VP3 junction of a Brazilian PeV-A1 strain shows that the N17/feces/PeV-A1/BRA/2021 strain belongs to subtype HPeV-1B. It is closely related to the Milan_16-227 strain from Italy (2016), with 96.9% nt identity (Figure 5).

## 4. Discussion

This study reveals that respiratory illness and gastroenteritis in children in the northwestern metropolitan area of São Paulo during spring 2021 were due to concurrent HBoV (respiratory symptoms) and NoV (gastrointestinal symptoms) infections. Additionally, these children were exposed to other enteric and respiratory pathogens, such as EV, PeV-A, and HAdV. Although mixed viral infections causing gastroenteritis or respiratory illness are globally documented, including in Brazil [24,25], studies on both conditions combined are relatively rare [2,26]. The comprehensive understanding of viral agents responsible for concurrent diarrheal and respiratory illnesses remains notably limited in Brazil. This investigation enhances the understanding of viral agents responsible for concurrent diarrheal and respiratory illnesses in Brazil, offering new perspectives for surveillance efforts.

HBoV infections in patients with influenza-like illness occur year-round, peaking in winter and spring, including in Brazil [6,27], supporting our data. In tropical regions like Brazil, NoV circulates consistently without a seasonal peak [8]. Globally, HBoV often co-infects with other enteric viruses, and studies in Brazil report frequent HBoV and NoV co-circulation [6,28]. However, consensus on the role of HBoV co-infections is difficult due to variations in study designs, inclusion criteria, and the extent of gastroenteric virus testing [28].

HBoV-1 was the only detected genotype, aligning with global findings and underscoring its widespread distribution and strong association with respiratory infections [6,27]. The genetic analysis revealed minimal variability (0.1% nt), consistent with previous studies [29], indicating limited genetic diversity. Despite this low variability, the HBoV-1 strains in this study formed distinct branches, suggesting they do not share a common origin and that there was no ongoing community outbreak of HBoV-1.

Molecular data from NoV strains suggest no outbreak in the studied population but rather an overlap of respiratory and enteric diseases. Three distinct NoV genotypes (GII.4_Sydney[P16], GII.4_Sydney[P31], and GII.2[P16]) were identified, indicating multiple sources of infection. These genotypes are predominant globally and in Brazil [30,31], in agreement with our findings. GII.2[P16] emerged during Japanese outbreaks in 2009–2010, spreading to Asia, Europe, and Australia [32,33,34,35], and reaching Brazil in 2017 [8]. GII.4_Sydney[P31] has been prevalent worldwide since 2012, associated with outbreaks, sporadic cases, and asymptomatic infections [36]. The GII.4_Sydney[P16] strain, identified in the US in 2015, also became globally dominant [8,37].

In addition to HBoV and NoV, children in Barueri were co-infected with other fecal and airborne viruses. Despite the presence of mixed infections, the gastroenteritis and respiratory symptoms were mild and similar to those observed in single viral infections. Additionally, it is important to note that the present study involved only an outpatient population treated at a pediatric emergency department, with no severe clinical outcomes or hospitalizations required. The identified HAdV-C6 is a rare type usually linked to respiratory infections [38], with sporadic cases in Brazil [39]. The detection of RSV and Flu A was expected due to the study period aligning with their seasonal occurrence [24].

The study detected two emerging viruses: CVA6 and PeV-A. Since 2008, CVA6 has been linked to a global rise in Hand, Foot, and Mouth Disease (HFMD), including outbreaks in Brazil since 2018 [40,41]. CVA6 has also been associated with central nervous system infections and severe acute respiratory illness [23,42,43]. The two children infected with CVA6 (patients N04 and N17) did not display typical HFMD symptoms, suggesting a link between CVA6 and respiratory symptoms, which is supported by the detection of CVA6 in the swab and feces of both patients, suggesting an ongoing infection. The Brazilian CVA6 strains N04 and N17 identified here exhibit distant genetic relationships. CVA6-N04 strains are genetically linked to those concurrently circulating in Brazil, while CVA6-N17 strains are associated with strains circulating in both Brazil and Asian countries [42,44]. Sub-lineage D3 is the main CVA6 sub-lineage worldwide, associated with HFMD, meningitis, and respiratory diseases [23,41,43].

PeV-A1 was detected in fecal samples from patient N17. PeV-A1 has been associated with both mild respiratory and gastrointestinal infections [45,46], supporting the data obtained here. The original PeV-A1 strain was identified in the USA in 1956 and has since spread globally [7], becoming the most common genotype in many countries [45,46]. Data on PeV-A in Brazil are limited, with little known about the predominant genotypes [47,48]. This study highlights the need for further investigation to understand PeV-A’s impact on the health of the Brazilian population, especially children.

No SARS-CoV-2 cases were detected despite the ongoing pandemic. Although COVID-19 vaccination for children under 5 had not begun, isolation measures were eased with daycare centers and schools reopening in Barueri [49], where isolation rates ranged from 28% to 48% during the study (https://www.saopaulo.sp.gov.br/coronavirus/isolamento/, accessed on 28 February 2025). This absence highlights the effectiveness of daycare centers’ preventive measures. Reports on HBoV during COVID-19 show varied patterns: some indicate increased rates, others decreased, and some unchanged pre- and post-pandemic [50,51,52]. Few studies in Brazil have investigated HBoV prevalence during the pandemic [53]. Interestingly, while enveloped viruses like Flu A/B and RSV nearly disappeared, non-enveloped viruses like HBoV, HAdV, and EV were still detected during the pandemic’s peak [52], aligning with our findings.

## 5. Conclusions

Our data indicate that HBoV and NoV caused acute respiratory disease in children ≤ 3 years old with diarrhea in northwestern Metropolitan São Paulo in 2021, confirming the overlap between respiratory and enteric diseases. Molecular data suggest no community outbreak, as HBoV and NoV strains showed distinct genotypes and do not share a common origin. The main limitation of this study was the small sample size, resulting from emergency room overcrowding during the COVID-19 outbreak, which led to early discharges and a reduced number of paired sample collections. This constraint impaired a more robust generalization of our findings. Therefore, future studies with larger patient cohorts are warranted to strengthen the validity of our data. Despite the small sample size, multiple viruses were identified, highlighting local viral diversity and high exposure to enteric viruses. Additionally, the cross-sectional design of the present investigation precluded conclusions regarding viral persistence, especially for pathogens like HBoV, which can be shed for prolonged periods [6]. A longitudinal study would be valuable to assess whether the viruses detected represent acute infections or prolonged shedding. These findings underscore the challenges of differential diagnosis due to overlapping symptoms and highlight the importance of syndromic surveillance in capturing the full scope of viral infections in similar clinical settings.

## Figures and Tables

**Table 1:** Clinical and demographic data from the 20 children with acute respiratory disease and diarrheic symptoms, Barueri, São Paulo, Brazil, spring 2021.

ID	Gender	Age	Onset Date	Collection Date	City	Signs and Symptoms	HBoV, Type	RVA	HAdV, Type	EV, Type	SARS-CoV-2	Flu A/B	RSV	HAstV	NoV, Group	NoV Strain	PeV-A, Type	Sample
N01	Male	1 year	August 8	August 30	Barueri	Diarrhea	+, NT	-	-	-	-	-	+	-	-		-	Swab
							-	-	-	-	-	-	+	-	+, GII	GII.4 Sydney[P16]	-	Feces
N02	Male	1 year	August 28	September 3	Barueri	Diarrhea, vomiting, coryza, cough, fever	+, NT	-	-	-	-	-	-	-	-		-	Swab
							-	-	-	-	-	-	-	-	+, GII	GII.4 Sydney[P16]	-	Feces
N03	Female	1 year	August 31	September 3	Carapicuiba	Diarrhea	+, NT	-	-	-	-	-	+	-	-		-	Swab
							+, NT	-	-	-	-	-	-	-	+, GII	NT	-	Feces
N04	Male	2 years	September 5	September 5	Barueri	Diarrhea, vomiting	+, HBoV-1	-	-	+, CVA6	-	-	-	-	-		-	Swab
							+, HBoV-1	-	-	+, CVA6	-	-	-	-	+, GII	GII.4 Sydney[P31]	-	Feces
N05	Female	1 year	September 5	September 6	Osasco	Diarrhea, cough, fever	+, HBoV-1	-	-	-	-	-	-	-	-		-	Swab
							+, HBoV-1	-	-	-	-	-	-	-	+, GII	GII.2[P16]	-	Feces
N06	Female	6 months	September 6	September 8	Osasco	Diarrhea, cough, vomiting	-	-	-	-	-	-	-	-	+, GII	GII.4 Sydney[P16]	+, NT	Feces
N07	Female	1 year	September 9	September 10	Barueri	Diarrhea	+, HBoV-1	-	-	-	-	-	-	-	+, GII	GII.4 Sydney[P16]	-	Feces
N08	Male	2 years	September 12	September 13	Barueri	Diarrhea, vomiting, coryza, fever, productive cough, abdominal pain	+, HBoV-1	-	-	-	-	-	-	-	+, GII	NT	-	Feces
N09	Male	3 years	September 20	September 21	Barueri	Diarrhea, vomiting	-	-	-	-	-	-	-	-	-		-	Swab
							+, NT	-	-	-	-	-	-	-	+, GII	GII.2[P16]	-	Feces
N10	Male	20 days	September 18	September 21	Barueri	Vomiting, fever	+, HBoV-1	-	-	-	-	-	-	-	-		-	Swab
							+, NT	-	-	-	-	-	-	-	-		-	Feces
N11	Female	1 year	September 12	September 13	Carapicuiba	Diarrhea, vomiting, coryza, cough, fever	-	-	-	-	-	-	-	-	-		-	Swab
							+, HBoV-1	-	+, C6	-	-	-	-	-	+, GII	GII.4 Sydney[P16]	-	Feces
N12	Male	2 years	September 20	September 22	Barueri	Diarrhea, vomiting, coryza, sore throat, abdominal pain	+, HBoV-1	-	-	-	-	-	-	-	-		-	Swab
							+, NT	-	-	-	-	-	-	-	+, GII	GII.4 Sydney[P16]	-	Feces
N13	Male	11 days	September 20	September 21	Barueri	Fever	+, NT	-	-	-	-	-	-	-	-		-	Swab
							+, NT	-	-	-	-	-	-	-	-		-	Feces
N14	Female	6 months	September 26	September 28	Carapicuiba	Diarrhea, cough	-	-	-	-	-	-	-	-	-		-	Swab
							-	-	-	-	-	-	-	-	-		-	Feces
N15	Male	1 year	October 2	October 8	Sao Paulo	Diarrhea, vomiting, coryza, fever, sneeze, productive cough, abdominal pain	+, HBoV-1	-	+, C6	-	-	-	-	-	-		-	Swab
							+, NT	-	-	-	-	-	-	-	+, GII	GII.2[P16]	-	Feces
N16	Female	1 year	October 7	October 9	Barueri	Diarrhea, Fever	+, NT	-	-	-	-	-	-	-	-		-	Swab
							+, NT	-	-	-	-	-	-	-	+, GII	GII.4 Sydney[P16]	-	Feces
N17	Female	5 months	October 11	October 11	Osasco	Diarrhea, vomiting	-	-	-	+, CVA6	-	-	-	-	-		-	Swab
							-	-	-	+, CVA6	-	-	-	-	+, GII	GII.4 Sydney[P16]	+, PeV-A1	Feces
N18	Male	1 year	October 14	October 17	Carapicuiba	Diarrhea, vomiting, coryza, sneeze, productive cough	+, NT	-	-	-	-	-	-	-	-		-	Swab
							-	-	-	-	-	-	-	-	+, GII	GII.4 Sydney[P16]	-	Feces
N19	Male	1 year	November 30	December 4	Itapevi	Diarrhea, coryza, productive cough, fever	-	-	-	-	-	-	-	-	-		-	Swab
							-	-	-	-	-	-	-	-	-		-	Feces
N20	Male	2 years	December 2	December 6	Carapicuiba	Diarrhea, vomiting, coryza, cough	-	-	-	-	-	Flu A	-	-	-		-	Swab
							-	-	-	-	-	-	-	-	-		-	Feces

NT: non-typed.

## Data Availability

Nucleotide sequences from this study were submitted to GenBank with accession numbers PP277716-PP277721 (HBoV-1), PP384164-PP384172 (GII.4_Sydney[P16]), PP384190 (GII.4_Sydney[P31]), PP386388-PP386390 (GII.2P[16]), PP429525-PP429526 (HAdV-C6), PP42952-PP429530 (CVA6), and PP496531 (PeV-A1).
